# Application of Dual Phase Imaging of ^11^C-Acetate Positron Emission Tomography on Differential Diagnosis of Small Hepatic Lesions

**DOI:** 10.1371/journal.pone.0096517

**Published:** 2014-05-09

**Authors:** Li Huo, Yonghong Dang, Jingqiao Lv, Haiqun Xing, Fang Li

**Affiliations:** Department of Nuclear Medicine, PET Center, Peking Union Medical College Hospital, Beijing, China; The University of Chicago, United States of America

## Abstract

**Objective:**

Previously we observed that dual phase ^11^C-acetate positron emission tomography (AC-PET) could be employed for differential diagnosis of liver malignancies. In this study, we prospectively evaluated the effect of dual phase AC-PET on differential diagnosis of primary hepatic lesions of 1–3 cm in size.

**Methods:**

33 patients having primary hepatic lesions with size of 1–3 cm in diameter undertook dual phase AC-PET scans. Procedure included an early upper-abdomen scan immediately after tracer injection and a conventional scan in 11–18 min. The standardized uptake value (SUV) was calculated for tumor (SUVT) and normal tissue (SUVB), from which ^11^C-acetate uptake ratio (as lesion against normal liver tissue, SUVT/SUVB) in early imaging (R1), conventional imaging (R2), and variance between R2 and R1 (ΔR) were derived. Diagnoses based on AC-PET data and histology were compared. Statistical analysis was performed with SPSS 19.0.

**Results:**

20 patients were found to have HCC and 13 patients had benign tumors. Using ΔR>0 as criterion for malignancy, the accuracy and specificity were significantly increased comparing with conventional method. The area under ROC curve (AUC) for R1, R2, and ΔR were 0.417, 0.683 and 0.831 respectively. Differential diagnosis between well-differentiated HCCs and benign lesions of FNHs and hemangiomas achieved 100% correct. Strong positive correlation was also found between R1 and R2 in HCC (r^2^ = 0.55, P<0.001).

**Conclusions:**

Dual phase AC-PET scan is a useful procedure for differential diagnosis of well-differentiated hepatocellular carcinoma and benign lesions. The dynamic changes of ^11^C-acetate uptake in dual phase imaging provided key information for final diagnosis.

## Introduction

Hepatocellular carcinoma (HCC) is the third most common cause of cancer related death in the world [1]. Many studies have reported significant benefit for patients if treatment was applied at early stage when HCC is small in size (less than 3 cm). Small HCC was defined as single cancerous nodule or two adjacent nodules of HCC having a total diameter less than 3 cm. Compared with larger HCC, most of small HCCs are isolated nodules with intact capsule. Histologically small HCC cancer cells are more unanimous in cell morphology with high level of differentiation, where positive correlation can be found between differentiation level and tumor size. Small HCC has significantly better prognosis than larger HCC after surgical resection [2].

^11^C-acetate positron emission tomography (AC-PET) has been proven to have high sensitivity in diagnosis of well-differentiated HCC [3], and be a useful alternative method to complement the limitation of FDG-PET for HCC diagnosis [4]–[7]. Since the normal liver tissue has a baseline uptake of acetate, AC-PET is not a sensitive measurement for tumor <1 cm. However, challenges also existed for AC-PET in differential diagnosis for hepatic lesions of 1–3 cm in diameter, due to increase of atypical features. Some benign liver lesions shared the same PET appearance as HCC, such as focal nodular hyperplasia (FNH) and hemangioma [8], [9]. Conventional AC-PET has been insufficient to complement FDG-PET for a better differential diagnosis [10], [11].

AC-PET conventional scan was routinely operated beginning 10–20 minutes after tracer injection [3], [4], by which tracer uptake of targeting lesion at a single time point was acquired and analyzed. Recently we reported a new method of PET scanning with dual-phase imaging, which was developed for a dynamic observation of the tracer uptake [12]. Though the finding in the report presented a successful case in identifying FNH, there was no statistical analysis and systematic characterization of dual-phase AC-PET imaging, which may have the potential to promote clinical differential diagnosis of liver lesion.

To further investigate the efficacy of dual phase AC-PET scan on differential diagnosis, particularly for small size liver lesions, we performed a prospective study that focused on 1–3 cm lesions that were suspected to be malignant. Our finding suggested that dual phase AC-PET imaging provided key marker that can be more valuable for differential diagnosis of small hepatic lesions than conventional imaging.

## Materials and Methods

### Patients

The study was approved by the Ethic Committee of Peking Union Medical College Hospital and the written informed consent was obtained from each patient. The patient inclusion criteria include: 1) patients must be 18 years old or above; 2) patients were capable of understanding the study and sign the informed consent; 3) patients had hepatic lesion of 1–3 cm in diameter by regular imaging measurement; and 4) diagnosed as malignancy by enhanced CT and/or MRI. Pregnant or breastfeeding patients were excluded from the study. A total of 33 patients aging 55.4±13 years old with liver occupying lesion, 27 males and 6 females, were enrolled into the study from January 2008 to October 2013. AC-PET scan was performed prior to surgical operation. Final diagnoses were made based on pathological results and tumors were graded using Edmondson-Steiner criteria [13].

### ^11^C-Acetate Synthesis/Preparation

^11^C-acetate was synthesized by transferring the ^11^C-CO_2_ generated by Siemens RDS111 cyclotron into a fully automated radiochemistry module based on the procedure described by Mitterhauser *et al*. [14] The chemical and radiochemical purities were both greater than 98%. The product was proved to be sterile by 24 h bacterial culture. Administration dosage per patient was 425-814MBq (3.7-4.4MBq/kg).

### PET scan

PET/CT scans were conducted on a Siemens Biography 64 True Point PET/CT scanner (Siemens Medical Solution, Inc.). A transmission scan was performed prior to ^11^C-acetate administration for attenuation correction. Immediately after the administration of ^11^C-acetate, an upper abdominal early phase PET data acquisition was performed for one bed position (emission scanning), covering the liver, spleen, part of the kidney and lower part of the heart. At 11–18 min after ^11^C-acetate administration, conventional scan was performed from pubic symphysis to cerebellum. Each scanning took 2 min per bed position.

PET images were reconstructed using the iterative OSEM algorithm. Images were analyzed by two independent nuclear medicine physicians who were blinded from the patient information.

### PET image analysis

The region of interest (ROI) was drawn automatically at 75% threshold of the maximum lesion count around the tumor. The standardized uptake value within the ROI on the lesions (SUVT) and normal liver tissue of same size as ROI (SUVB) were obtained for quantitative analysis. Tracer uptake ratio by tumor R was obtained (R =  SUVT/SUVB) for the early imaging R1 and conventional imaging R2. The variance from R1 to R2 was calculated as ΔR (ΔR  =  R2-R1).

### Statistical analysis

Data were statistically analyzed with SPSS19.0 (IBM, USA). Comparison among multiple groups was performed with one way ANOVA. Pearson correlation or Chi square test was analyzed for correlation between R1 and R2, and two-tailed probability at 0.05 was taken as significant level. A receiver operating characteristic (ROC) curve was used for evaluation of diagnostic criteria.

## Results

Patients, who had a hepatic lesion of 1–3 cm in diameter and malignancy was diagnosed by enhanced CT and/or MRI, were enrolled in the study. Dual-phase AC-PET scanning was performed on all patients prior to surgical operation. Data from individuals, including tumor size, PET scan results, and pathological diagnosis, were shown in table 1. About 39.4% (13/33) were benign lesions and the other 60.6% (20/33) were malignant as diagnosed histologically. All malignant tumors were HCCs, among which 10 were highly differentiated, 6 were moderate differentiated, and 4 were poorly differentiated lesions. Within the benign lesions, we found multiple different lesions including 4 FNHs, 2 hemangiomas, 1 adenoma, 1 dysplasia nodule, 1 reactive hyperplasia lymph node, and 4 inflammatory lesions (Table 1).

**Table 1:** General information of tumor size, PET results and pathological diagnosis.

#	Size (cm)	R1	R2	ΔR	Pathological diagnosis*
1	1.0	1.15	1.97	0.82	HCC-I
2	2.0	1.05	1.41	0.36	HCC-I
3	2.5	2.50	2.94	0.44	HCC-I
4	2.0	2.13	3.08	0.95	HCC-I
5	2.5	1.56	1.82	0.26	HCC-I
6	1.1	1.43	1.71	0.28	HCC-I
7	2.0	1.61	2.32	0.71	HCC-I
8	2.0	1.92	2.77	0.85	HCC-I
9	1.9	1.86	1.00	−0.86	HCC-I
10	3.0	1.30	1.82	0.52	HCC-I
11	1.3	1.50	2.10	0.60	HCC-II
12	1.5	1.06	1.09	0.03	HCC-II
13	3.0	1.51	2.10	0.59	HCC-II
14	3.0	1.48	1.50	0.02	HCC-II
15	1.0	1.18	1.22	0.04	HCC-II
16	2.5	1.01	1.17	0.16	HCC-II
17	2.0	1.80	1.26	−0.54	HCC-III
18	1.5	1.00	1.00	0	HCC-III
19	1.5	1.00	1.00	0	HCC-III
20	1.0	1.00	1.00	0	HCC-III
21	2.5	2.22	1.00	−1.22	B (FNH)
22	2.0	3.30	1.30	−2.00	B (FNH)
23	3.0	1.63	1.36	−0.27	B (FNH)
24	2.0	1.90	1.41	−0.49	B (FNH)
25	2.1	1.40	0.40	−1.00	B (hemangioma)
26	1.8	1.57	1.18	−0.39	B (hemangioma)
27	2.0	1.65	2.53	0.88	B (adenoma)
28	2.3	2.29	1.69	−0.60	B (dysplasia nodule)
29	1.2	1.39	1.61	0.22	B (reactive hyperplasia lymph node)
30	2.6	1.00	1.00	0	B (inflammation)
31	1.0	1.00	1.00	0	B (inflammation)
32	2.0	1.50	1.47	−0.03	B (inflammation)
33	1.7	0.48	0.40	−0.08	B (inflammation)

* I, Edmondson-Steiner grade I and II; II, Edmondson-Steiner grade III; and III, Edmondson-Steiner grade IV; B, benign.

### Effect of dual phase AC-PET on HCC diagnosis

**Figure 1:**
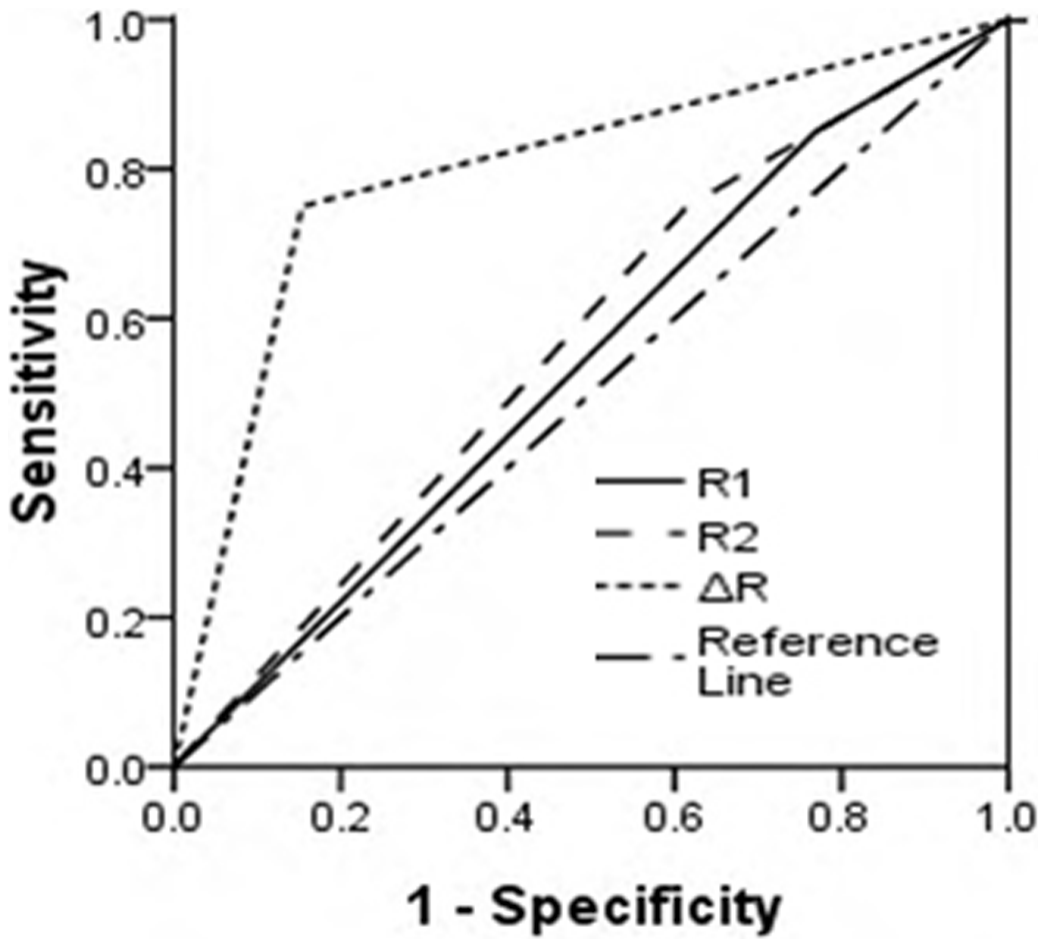
The area under the curve of ROC for R1, R2 and ΔR. AUC for R1 = 0.417, SE = 0.109; AUC for R2 = 0.683, SE = 0.095; AUC for ΔR = 0.831, SE = 0.077. The diagnostic accuracy of the ΔR was statistically significantly better than that of R1 and R2 (P<0.05).

Three criteria were implemented for liver malignancy based on dual phase AC-PET scan: early imaging positive (R1>1, by which positive means stronger signal in tumor than normal liver tissue), conventional imaging positive (R2>1) and a positive variance from R1 to R2 (ΔR>0). Diagnosis specificity and sensitivity were calculated and summarized in table 2. Early phase imaging alone was proved to have insufficient sensitivity, specificity and accuracy for HCC diagnosis. However, with the dynamic changes (ΔR) as criteria, we observed substantial improvement of PET diagnosis *vs.* R2 in accuracy (78.8% *vs.* 63.6%), specificity (84.6% *vs*. 38.5%), negative predictive value (68.8% *vs*. 55.6%), and positive predictive value (88.2% *vs.* 66.7%) (Table 2). A receiver operating characteristic (ROC) curve was generated for R1, R2, and ΔR and the areas under ROC curves (AUC) were 0.417, 0.683 and 0.831 respectively (Figure 1). Significant difference was found between the AUC of R2 and ΔR (P<0.05).

**Table 2:** Statistical analysis of the R1, R2, and ΔR as criteria for hepatic lesion diagnosis.

Diagnosis Criteria	Sensitivity	Specificity	Accuracy	Negative predictive value	Positive predictive value
R1>1	85.0	23.1	60.6	50.0	63.0
R2>1	80.0	38.5	63.6	55.6	66.7
ΔR>0	75.0	**84.6**	**78.8**	**68.8**	**88.2**

### Differential diagnosis of FNH and hemangioma from well differentiated HCC

**Figure 2:**
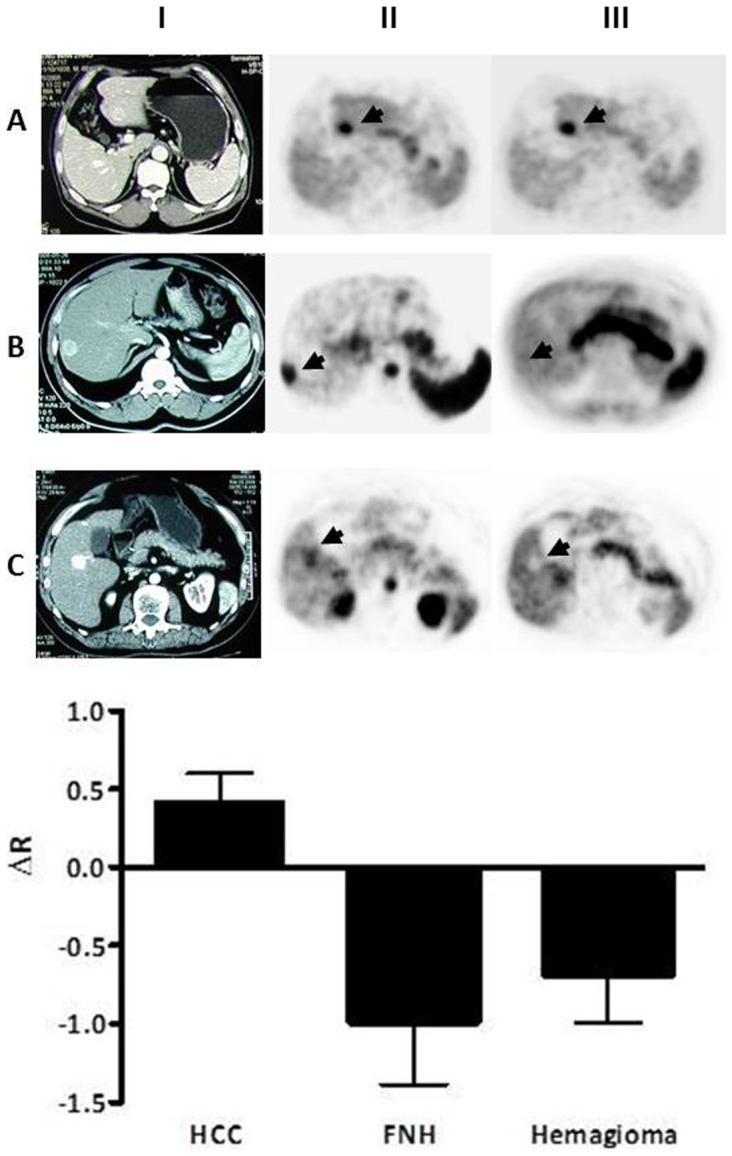
Comparison of malignant and benign lesions in dual phase imaging. Upper panel: Images to illustrate differential diagnosis of liver lesion with dual AC-PET imaging. Column I is the images from enhanced CT for the confirmation of elevated arterial perfusion. Column II and III are images from early stage and conventional imaging respectively. Row A is from a case of well-differentiated HCC, R1 = 2.13, R2 = 3.08. Row B is from a case of FNH, R1 = 2.22, R2 = 1.00. Row C is a case of hepatic hemangioma, R1 = 1.40, R2 = 0.40. Lower panel: Characteristic changes between R1 and R2 in HCC, FNH and hemangioma. Well-differentiated HCC showed a positive value of ΔR while the benign lesions exhibited negative ΔR.

Ten well-differentiated HCCs (Edmondson-Steiner grade I and II), four FNHs and two hemangiomas were diagnosed in the study. Both HCC and FNH showed positive uptake in early phase and conventional imaging. The dynamic change of tracer uptake increased in 9 out of 10 HCCs (ΔR = 0.43±0.24 mean±SD) but decreased in FNHs (ΔR = −0.99±0.78). Two small hemangioma lesions also exhibited substantial negative value of ΔR (−0.39 and −1.00 respectively) (Figure 2).

### Comparison of early imaging with conventional imaging

Positive R1 values in early imaging were obtained in 27 patients, fluctuating between 1.01 and 3.30 among which the highest number came from a case of FNH and lowest number was found from a HCC. 24 of them remained being positive in conventional imaging with R2  =  1.09–3.08, where 3.08 came from a case of well-differentiated HCC and 1.09 was from a moderate differentiated HCC. Increases of ^11^C-acetate uptake during the conventional imaging were observed in 17 of the 27 positive cases (ΔR>0), including 15 cases of HCC, 1 adenoma, and 1 reactive hyperplasia lymph node. The rest 10 cases were found to have a reduced tracer uptake in conventional imaging (ΔR<0).

For the 6 cases with negative early imaging (R1≤1), all conventional imaging remained the same or reduced value (ΔR≤0). Five cases showed same uptake as normal liver tissue in both early phase and conventional imaging (Table 1).

**Figure 3:**
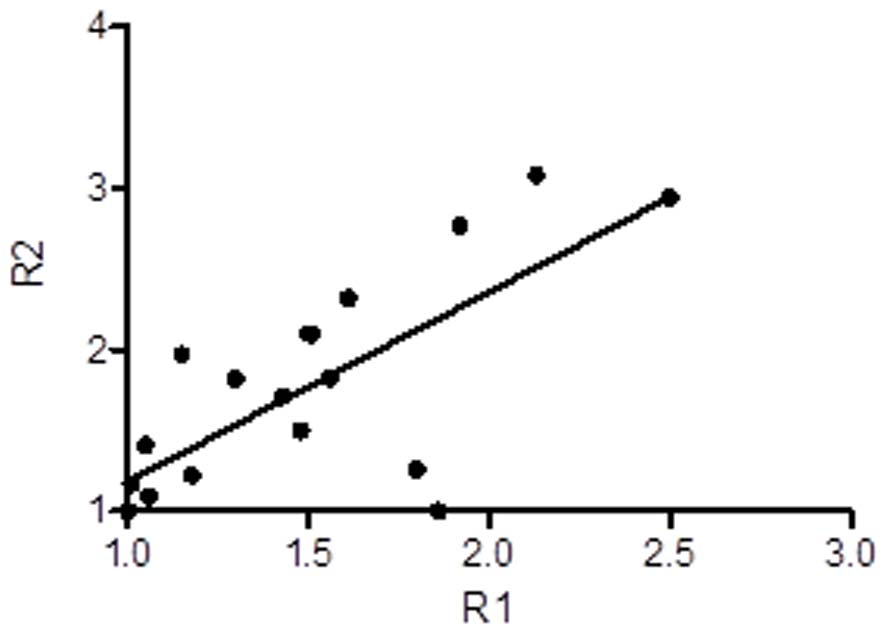
Correlation of R1 and R2 from dual-phase PET imaging. The tracer uptake ratio from early phase imaging (R1) and conventional imaging (R2) of 20 HCC patients were plotted and linear regression was generated. r^2^ = 0.55 and P<0.01

Pearson correlation test of early phase uptake ratio R1 with conventional imaging uptake ratio R2 in HCC was shown in Figure 3. Significant positive correlation was found between R1 and R2 among HCC cases where r^2^ = 0.55 and P<0.01. No correlation was found in benign lesions.

## Discussion

Differential diagnosis of small liver lesions (≤3 cm) has been challenging for all means of measurement [15], [16]. Large biopsy or surgical resection remained golden standard of final diagnosis. In this study, application of dual phase imaging of AC-PET was especially helpful in the differential diagnosis of FNH and small hemangioma from malignant lesions. By conventional acquisition, FNH exhibited to be positive, which was difficult to be distinguished from well differentiated HCC [10], and hemangioma was even more challenging for PET diagnosis, for its heterogeneity could be easily confused with either well differentiated HCC (positive R2) or poor differentiated HCC (negative R2) [17], [18]. Addition of early phase imaging would provide crucial information of tracer variance that both HCC and benign lesion have distinct different patterns. Both FNH and small hemangioma are hyper vascularized lesions and exhibited positive tracer uptake ratio in the early phase imaging, followed by an immediate decline of signal [12]. Therefore FNH and small hemangioma showed a significant reduction while well-differentiated HCC demonstrated increasing uptake over time. Despite of limited case number, we have proven of concept that dual phase imaging could be a useful method for differential diagnosis of HCC and benign liver lesions. This was particular important in preventing patients from unnecessary aggressive treatment for benign lesions.

PET provides functioning image of the targeted cells; it is reasonable to assume that more valuable data can be generated by dynamic observation. Our data demonstrated that specificity and accuracy were significantly increased when ΔR was used as a differential diagnosis criterion comparing with conventional AC-PET. As reported previously, tracer activity rapidly rises in HCC within two minutes after a bolus IV injection and followed by a gradual increase over time due to high vascularization [19]. While this was observed in most cases in our study, 3 of the 20 HCCs had R1≤1, all of which are poorly differentiated HCCs. Although HCC is a highly angiogenic cancer, tumor vessels have an abnormal blood flow and may be excessively leaky. In turn, this may leads to hypovascular areas and severe hypoxia, which is typically less favorable for AC uptake [20]. Byam *et al.* reported that microvasculature in tumor is less dense than that in normal liver [21]. Yang *et al.* found that the degree of vascularization in HCC may be correlated with tumor differentiation [22], which is in consistent with our finding in this study.

We found that the tracer uptake at conventional imaging (R2) of HCC was highly correlated with that in the early phase (R1). Most of the HCCs (except two cases) exhibited increases of tracer accumulation in the conventional imaging, where the range of increase varied between 1–71% of R1 value. This wide range might be due to the characteristics of the tumor mess, as well as the acquisition starting point of R2 that was taken from 11–18 min after tracer injection. However, our evaluation on acquisition starting time point of R2 uptake didn't show significant difference within the time period (data not shown).

No correlation was found between R1 and R2 in benign lesions, part of the reason might due to the variety of diseases and small case number of each type. Surprisingly, it is noticeable that none of the negative early imaging turned positive in conventional imaging. This might be an indication of 1) the crucial role of perfusion of tumor for subsequent metabolic accumulation of ^11^C-acetate; 2) poorly differentiated tumor that had a decreased uptake due to poor vascularization or low activity of ACSII, a key enzyme for acetate metabolism in tumor cells [23]; and 3) benign lesion. We have observed in these cases that prolonged acquisition in conventional imaging would not elevate the tracer uptake. For these cases, FDG-PET should be a preferred method for diagnosis.

The goal of this study was intended to clarify the superiority of dual-phase AC-PET over conventional AC-PET imaging in differential diagnosis of liver lesion. One limitation of the study is that some poorly differentiated HCCs also showed similar manifestation as FNH and hemangioma. Combination of both FDG and dual phase AC PET could be applied to resolve this issue. The limited number and variety of benign lesion types prevented us from drawing a conclusion about application of dual phase AC-PET on differentiation of benign lesions.

In summary, AC-PET dual-phase imaging is a simple method through which well-differentiated HCC and certain small size benign lesions, such as FNH and hemangioma, can be differentiated. Application of dynamic change of tracer uptake significantly improved sensitivity and accuracy of AC-PET for HCC diagnosis. It would be a simple and efficient adjustment to change conventional AC-PET scan to dual-phase scan.
